# Development and validation of a 14-CpG DNA methylation signature and drug targets for prognostic prediction in breast cancer

**DOI:** 10.3389/fmed.2025.1548726

**Published:** 2025-03-19

**Authors:** Bao-xing Tian, Zhi-xi Yu, Xia Qiu, Li-ping Chen, Yu-lian Zhuang, Qian Chen, Yan-hua Gu, Meng-jie Hou, Yi-fan Gu

**Affiliations:** ^1^Hongqiao International Institute of Medicine, Tongren Hospital, Shanghai Jiao Tong University School of Medicine, Shanghai, China; ^2^Shanghai Key Laboratory of Tissue Engineering, Department of Plastic and Reconstructive Surgery, Shanghai 9th People’s Hospital, Shanghai Jiao Tong University School of Medicine, Shanghai, China; ^3^Department of Breast Surgery, Tongren Hospital, Shanghai Jiao Tong University School of Medicine, Shanghai, China; ^4^Department of Nursing, Tongren Hospital, Shanghai Jiao Tong University School of Medicine, Shanghai, China

**Keywords:** breast cancer, prognostic model, DNA methylation, biomarker, TCGA, GEO

## Abstract

**Background:**

Breast cancer (BC) is the most prevalent cancer among women and a leading cause of cancer-related deaths worldwide. Emerging evidence suggests that DNA methylation, a well-studied epigenetic modification, regulates various cellular processes critical for cancer development and progression and holds promise as a biomarker for cancer diagnosis and prognosis, potentially enhancing the efficacy of precision therapies.

**Methods:**

We developed a robust prognostic model for BC based on DNA methylation and clinical data from The Cancer Genome Atlas (TCGA) and Gene Expression Omnibus (GEO). We analyzed the association of the model with clinicopathological features, survival outcomes, and chemotherapy drug sensitivity.

**Results:**

A set of 216 differentially methylated CpGs was identified by intersecting three datasets (TCGA, GSE22249, and GSE66695). Using univariate Cox proportional hazard and LASSO Cox regression analyses, we constructed a 14-CpG model significantly associated with progression-free interval (PFI), disease-specific survival (DSS), and overall survival (OS) in BC patients. Kaplan–Meier (KM) survival analysis, receiver operating characteristic (ROC) analysis, and nomogram validation confirmed the clinical value of the signature. The Cox analysis showed a significant association between the signature and PFI and DSS in BC patients. KM analysis effectively distinguished high-risk from low-risk patients, while ROC analysis demonstrated high sensitivity and specificity in predicting BC prognosis. A nomogram based on the signature effectively predicted 5- and 10-year PFI and DSS. Additionally, combining our model with clinical risk factors suggested that patients in the I–II & M^+^ subgroup could benefit from adjuvant chemotherapy regarding PFI, DSS, and OS. Gene Ontology (GO) functional enrichment and KEGG pathway analyses indicated that the top 3,000 differentially expressed genes (DEGs) were enriched in pathways related to DNA replication and repair and cell cycle regulation. Patients in the high-risk group might benefit from drugs targeting DNA replication and repair processes in tumor cells.

**Conclusion:**

The 14-CpG model serves as a useful biomarker for predicting prognosis in BC patients. When combined with TNM staging, it offers a potential strategy for individualized clinical decision-making, guiding personalized therapeutic regimen selection for clinicians.

## Introduction

Female breast cancer (BC) has emerged as the most commonly diagnosed cancer among women globally, surpassing lung cancer, with an estimated 2.3 million new cases (1). Despite significant advancements in diagnostic and therapeutic modalities, BC remains a formidable health challenge, contributing to substantial mortality worldwide, with an estimated 685,000 deaths in 2020 (1, 2). The complexity of BC is underscored by its heterogeneous nature, characterized by diverse molecular profiles and histological types. This heterogeneity profoundly influences clinical responses to treatment modalities such as chemotherapy, hormonal therapy, radiotherapy, and more recently, immunotherapy and targeted therapy (3–5).

The classification of BC into molecular subtypes—luminal A, luminal B, HER2-enriched, and triple-negative BC (TNBC)—based on biomarkers like estrogen receptors (ER), progesterone receptors (PR), and human epidermal growth factor receptor 2 (HER2), directs therapeutic strategies. Systemic therapies are tailored accordingly: endocrine therapy for luminal BC, HER2-targeted therapies combined with chemotherapy for HER2-enriched BC, and chemotherapy for TNBC (5). However, resistance mechanisms often limit the efficacy of these treatments in a subset of patients.

The advent of precision medicine has revolutionized BC research, emphasizing the importance of predictive biomarkers in guiding treatment decisions. Tools such as Oncotype DX, MammaPrint, and EndoPredict aid in selecting patients who benefit most from specific therapies, thereby minimizing unnecessary overtreatment (6, 7). Nevertheless, the utility of current biomarkers is limited to certain patient subsets, necessitating the identification of novel prognostic biomarkers to refine treatment strategies and improve patient outcomes.

Epigenetics, focusing on modifications such as DNA methylation, histone modification, and non-coding RNA regulation, has emerged as a crucial area in BC research (8). DNA methylation, a well-studied epigenetic modification, regulates various cellular processes critical for cancer development and progression (9). Aberrant DNA methylation patterns, including hypermethylation of tumor suppressor genes, contribute significantly to BC pathogenesis (10). Moreover, DNA methylation alterations serve as promising biomarkers for cancer diagnosis and prognosis, including in non-invasive liquid biopsy approaches for TNBC (11). Integrating these epigenetic insights into clinical practice holds immense promise for advancing precision oncology (12). Recent advancements in genomic databases such as The Cancer Genome Atlas (TCGA) and Gene Expression Omnibus (GEO) provide valuable resources for studying cancer biology. These platforms facilitate comprehensive genomic and epigenomic analyses to identify novel biomarkers associated with cancer prognosis and therapeutic responses (13, 14). Leveraging data from TCGA and GEO, researchers can explore DNA methylation profiles to uncover unique signatures that may serve as biomarkers associated with disease progression in BC.

This study aims to identify a distinct DNA methylation signature associated with BC progression using TCGA and GEO databases. We intend to develop a survival nomogram incorporating this signature to enhance prognostic accuracy and guide personalized therapeutic interventions. Additionally, our investigation will examine the molecular characteristics of BC subgroups and assess drug sensitivity based on the Genomics of Drug Sensitivity in Cancer (GDSC) database, offering insights into therapeutic response prediction (15). Recent studies have developed several DNA methylation-based prognostic models for BC, including a 7-DNA methylation signature and a 6-gene prognostic signature based on differential DNA methylation, among others (16–18). While these models have provided valuable insights, they are often limited by poor reproducibility, a lack of multi-omics integration, and limited clinical applicability. In contrast, our 14-CpG model demonstrates several key advantages, including higher accuracy and reproducibility across both the training and validation sets, bolsters confidence in its prognostic potential. Additionally, our investigation can elucidate molecular characteristics and assess drug sensitivity across BC subgroups, offering insights into therapeutic response prediction.

## Methods

### Dataset selection

DNA methylation, RNA-seq, and clinical data were obtained from The Cancer Genome Atlas (TCGA) Genomic Data Commons portal (GDC, https://cancergenome.nih.gov/). Specifically, level 3 DNA methylation and RNA-seq data, along with corresponding clinical information, were downloaded for analysis. The BC dataset from TCGA was last updated on October 29, 2021, and all results presented here are based on this data. Additionally, two DNA methylation array datasets were collected from the Gene Expression Omnibus (GEO) for validation: GSE22249 (19) (114 tumor and 8 normal samples) and GSE66695 (80 tumor and 40 normal samples). The array platforms for GSE22249 and GSE66695 were Illumina HumanMethylation27 BeadChip (GPL8490) and Illumina HumanMethylation450 BeadChip (GPL13534), respectively.

### CpG identification

The DNA methylation levels for CpG sites were represented as *β*-values, calculated as:


$$\beta =\frac{\text{Intensity}\;\text{of}\;\text{the}\;\text{methylated}\;\text{allele}\;\left(M\right)}{\begin{array}{l} \text{Intensity}\;\text{of}\;\text{the}\;\text{unmethylated}\;\text{allele}\;\left(U\right)+\\ \text{Intensity}\;\text{of}\;\text{the}\;\text{methylated}\;\text{allele}\;\left(M\right)+100 \end{array}}$$

These *β*-values range from 0 (no methylation) to 1 (full methylation). Methylation data from TCGA were processed and merged using Strawberry Perl software (20). For RNA-seq data, normalization was performed using the “Normalize Quantiles” function in the edgeR package (R). Differential methylation analysis was conducted using the “limma” and “ggvenn” R packages, applying the Wilcoxon test to detect methylation differences between tumor and normal samples with *p*-values adjusted using the false discovery rate (FDR) method.

### Model development

A prognostic risk model was developed by analyzing DNA methylation data from 1,050 BC patients in TCGA, for whom clinical features and methylation data were available. Based on the 216 common differentially methylated CpGs identified by intersecting three datasets (TCGA, GSE22249, and GSE66695), we identified a total of 16 differentially methylated CpGs with prognostic value using univariate Cox proportional hazards models (*p*-value ≤0.05). To select a more compact signature, the 16 most prognostically significant CpGs were then further analyzed using multivariate Cox regression, with a stepwise backward elimination approach (entry *p*-value ≤0.05, removal *p*-value ≥0.10). Then, 14 of the 16 prognosis-related CpGs were determined by LASSO Cox regression analysis to constitute the optimal prognostic model for predicting PFI, and the risk score for each patient was calculated using the formula:


$$\text{Risk}\;\text{score}=\sum \left(\exp_{n}\times \beta_{n}\right)$$

where Exp*_n_* is the *β*-value of each CpG, and *β_n_* is the corresponding coefficient. Based on the calculated risk scores, patients were classified into low-risk or high-risk groups. Model accuracy was assessed using the area under the curve (AUC) from receiver operating characteristic (ROC) curve analysis, and Kaplan–Meier (KM) survival analysis was conducted to assess the survival differences between these groups. The model was validated using the GSE22249 dataset.

### Validation and nomogram construction

Independent prognostic predictors, identified via univariate and multivariate Cox regression, were incorporated into a nomogram using the R packages “rms,” “foreign,” and “survival.” The nomogram was designed to predict 5-year and 10-year progression-free interval (PFI), disease-specific survival (DSS), and overall survival (OS). Calibration plots were generated to assess the accuracy of the nomogram predictions. AUC and KM analysis were also applied to validate the nomogram’s prognostic capability.

### Bioinformatics analysis

Differential gene expression between the high-risk and low-risk groups was performed using the “limma” R package, resulting in 7,537 differentially expressed genes (DEGs). Gene Ontology (GO) and Kyoto Encyclopedia of Genes and Genomes (KEGG) pathway analyses were conducted to identify enriched biological processes and signaling pathways using the R packages “clusterProfiler,” “org.Hs.eg.db,” “enrichplot,” “ggplot2,” and “graphlayouts.” To gain further insights into the potential clinical applications of our CpG signature, we explored the tumor-infiltrating immune cells landscape using the CIBERSORT algorithm, which allows for the estimation of the relative abundance of immune cell subtypes in breast cancer samples. This analysis helps us understand the potential role of the immune microenvironment in modulating the prognostic value of our signature (21). Additionally, drug sensitivity analysis was performed using the Genomics of Drug Sensitivity in Cancer (GDSC) database,^1^ which provides IC50 values for a wide range of anticancer drugs across multiple cancer cell lines (15). To identify potential drugs for the low-risk and high-risk groups, patients were stratified into two groups based on their CpG-based risk scores. We performed a drug sensitivity analysis using the “oncoPredict” R package based on data from the GDSC database (15, 22, 23). Drugs showing significant differential sensitivity between the groups were identified as potentially effective for each risk group.

### Statistical analysis

Statistical analyses were performed using SPSS (version 25.0), Strawberry Perl (version 5.30.0.1), and R (version 3.6.1, 4.1.0, and 4.30). Statistical significance was defined as a two-sided *p*-value or adjusted *p*-value ≤0.05. The Wilcoxon test was used for differential analysis of methylated CpGs and DEGs between groups. The prognostic CpGs identified by univariate Cox regression (*p*-value ≤0.05) were further analyzed using LASSO Cox regression, with the final prognostic model developed based on the 14 most significant CpGs. Patients were classified into risk subgroups based on the calculated risk scores, and correlation analyses were conducted between clinicopathological features and these risk subgroups. The prognostic performance of the model was evaluated using ROC curves, and survival curves were generated using Kaplan–Meier analysis with the log-rank test. Chi-square tests were applied to assess the correlation between clinical features and risk subgroups.

## Results

### Identification of prognosis-related CpGs

**Figure 1:**
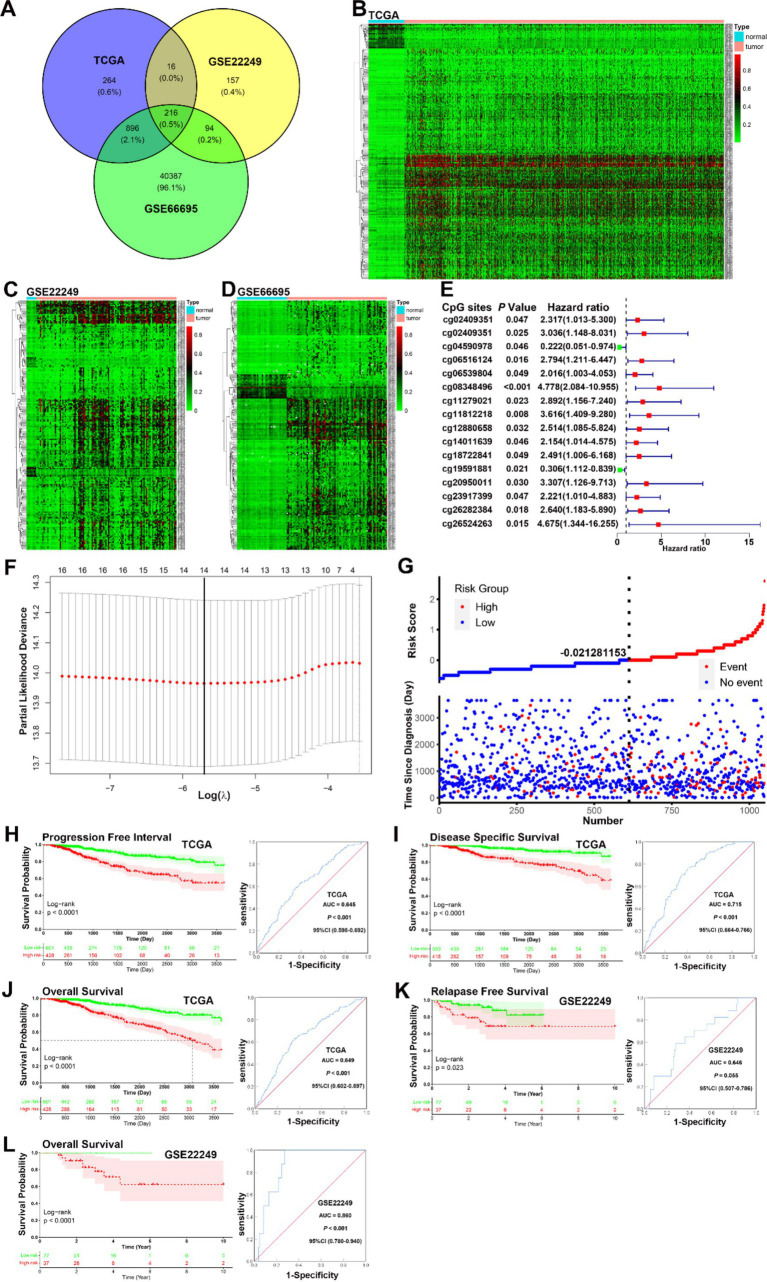
Identification of prognosis-related CpGs in BC. **(A)** A Venn diagram of the intersection of the 216 differential CpGs sets. **(B–D)** A heatmap plot of the methylation levels of 216 differential CpGs between the normal and tumor tissues in TCGA, GSE22249, and GSE66695. **(E)** A forest plot of 16 CpGs with prognostic value identified by univariate Cox regression analysis in the TCGA dataset. **(F)** LASSO regression analysis showed that 14 of the 16 prognosis-related CpGs were determined by LASSO Cox regression analysis to constitute the optimal prognostic model for PFI risk in BC patients. **(G)** The distribution and survival status of BC patients with different risk scores showed patients with high-risk scores tended to have poorer clinical outcomes compared to those with low-risk scores. **(H–J)** KM analysis indicated that patients in the low-risk group had significantly longer PFI, DSS, and OS in the TCGA datasets (all *p* < 0.0001). ROC curve analysis showed that the prognostic signature had good sensitivity and specificity for predicting PFI, DSS, and OS in the TCGA dataset (PFI, AUC = 0.645, 95% CI 0.598–0.692, *p* < 0.001; DSS, AUC = 0.715, 95% CI 0.664–0.766, *p* < 0.001; OS, AUC = 0.649, 95% CI 0.602–0.697, *p* < 0.001). **(K–L)** KM analysis indicated that patients in the low-risk group had better RFS and OS in the GSE22249 dataset (RFS, *p* = 0.023; OS, *p* < 0.001), ROC curve analysis confirmed that the prognostic signature also had good sensitivity and specificity for predicting RFS and OS in the GSE22249 dataset (RFS, AUC = 0.646, 95% CI 0.507–0.786, *p* = 0.055; OS, AUC = 0.860, 95% CI 0.780–0.940, *p* < 0.001).

Using the R packages “limma” and “ggvenn,” 216 common differentially methylated CpGs were identified by intersecting three datasets (TCGA, GSE22249, and GSE66695). The Venn diagram represents this intersection (Figure 1A), and the methylation expression levels are shown in heatmap plots (Figures 1B–D). Univariate Cox proportional hazard analysis identified 16 CpGs with prognostic value in the TCGA dataset, and these results are presented in a forest plot (Figure 1E). These findings provide a foundation for identifying key DNA methylation markers in BC that are associated with prognosis, potentially aiding in risk stratification and patient management.

### Construction of risk model based on prognosis-related CpGs

Based on univariate Cox proportional hazard analysis, 14 of the 16 prognosis-related CpGs were determined by LASSO Cox regression analysis to constitute the optimal prognostic model for predicting progression-free interval (PFI) risk in BC patients (Figure 1F). The risk scores were calculated using the formula: 2.79589 × 10^−4^ × cg02409351 + 2.450772 × 10^−4^ × cg03943081 − 1.372818 × 10^−4^ × cg04590978 + 2.963819 × 10^−4^ × cg06516124 + 6.293556 × 10^−5^ × cg06539804 + 5.488809 × 10^−4^ × cg08348496 + 5.200844 × 10^−4^ × cg11279021 + 7.529697 × 10^−5^ × cg12880658 + 9.462544 × 10^−5^ × cg14011639 − 9.566690 × 10^−4^ × cg19591881 + 2.912446 × 10^−4^ × cg20950011 + 5.356736 × 10^−4^ × cg23917399 + 2.892131 × 10^−4^ × cg26282384 − 1.212399 × 10^−4^ × cg26524263. Using the ROC curve and the distance on the curve, −0.021281153 was determined as the cut-off point (Table 1). Patients were then grouped into high-risk (*N* = 428) and low-risk (*N* = 601) categories. Patients with high-risk scores tended to have poorer clinical outcomes compared to those with low-risk scores (Figure 1G). KM analysis indicated that patients in the low-risk group had significantly longer PFI, disease-specific survival (DSS), and overall survival (OS) in the TCGA datasets (all *p* < 0.0001, Figures 1H–J). ROC curve analysis showed that the prognostic signature had good sensitivity and specificity for predicting PFI, DSS, and OS in the TCGA dataset (PFI, AUC = 0.645; DSS, AUC = 0.715; OS, AUC = 0.649, all *p* < 0.001) (Figures 1H–J). Although KM analysis indicated that patients in the low-risk group had better relapse-free survival (RFS) and OS in the GSE22249 dataset (RFS, *p* = 0.023; OS, *p* < 0.001), ROC curve analysis confirmed that the prognostic signature also had good sensitivity and specificity for predicting RFS and OS in the GSE22249 dataset (RFS, AUC = 0.646, *p* = 0.055; OS, AUC = 0.860, *p* < 0.001) (Figures 1K,L). Both KM and ROC analyses validate the prognostic power of the 14-CpG risk signature, with strong potential for predicting survival outcomes in multiple independent datasets. This reinforces its utility in clinical settings for stratifying patients based on prognosis. The 14-CpG risk model provides an effective method for predicting the prognosis of BC patients, with the high-risk group showing worse clinical outcomes. This model could be used to guide personalized treatment strategies for patients with BC.

**Table 1:** ROC curve for various cut-off levels of the risk score.

The risk score	Sensitivity	Specificity	DOC
−0.0354	0.640	0.596	0.5411
−0.0230	0.625	0.611	0.5407
−0.0224	0.625	0.612	0.5399
−0.0213	0.625	0.613	0.539 (cut-off)
−0.0156	0.618	0.617	0.5411

^*^AUC: 0.760 (95% CI: 0.719—0.800), *p* < 0.001. DOC, distance on curve equaling square root of (1 − Sen)^2^ + (1 − Spe)^2^; AUC, area under curve.

### Demographic and clinical, pathologic characteristics

A total of 1,029 BC cases recorded in TCGA were used to construct the prognosis model. The median patient age was 58 years (ranging from 26 to 90 years), with median PFI, DSS, and OS of 767 days, 825 days, and 821 days, respectively. The 10-year PFI rate was 87.7%, the 10-year DSS rate was 92.9%, and the 10-year OS rate was 87.6%. Tumor size, lymph node status, and metastasis status were defined according to the Eighth Edition American Joint Committee on Cancer (AJCC) Staging Manual (24), and molecular subtypes (PAM50) were derived from Thorsson et al. (25). In the molecular subgroup, the proportions of normal and luminal A patients in the low-risk group were significantly higher than in other subgroups, especially HER2 and basal subgroups, in the TCGA dataset (*χ*^2^ = 79.974, *p* < 0.001). Higher tumor size was associated with a higher proportion of patients in the high-risk group (tumor size status, *χ*^2^ = 14.271, *p* = 0.001). No statistically significant differences were found in age, lymph node status, and metastasis status subgroups (age, *χ*^2^ = 0.460, *p* = 0.100; lymph node status, *χ*^2^ = 4.297, *p* = 0.117; metastasis status, *χ*^2^ = 2.924, *p* = 0.087). The demographic and clinical, pathologic characteristics of BC patients are shown in Table 2. The demographic and clinical characteristics of the patients suggest that tumor size and molecular subtypes are closely associated with the risk classification. These findings underscore the relevance of integrating molecular features and clinical parameters when assessing BC prognosis.

**Table 2:** Demographic and clinical, pathologic characteristics of the patient with BC.

Variable	Total	Group		*c* ^2^	*p*-value
		Low risk	High risk		
	*n* = 1,029	*n* = 601	*n* = 428		
Age, years					
<41	95	63	32	4.600	0.100
41–60	475	284	191		
>60	459	254	205		
Molecular subtype					
Normal	130	86	44	79.974	<0.001
Luminal A	481	338	143		
Luminal B	177	85	92		
HER2	70	29	41		
Basal	171	63	108		
Tumor size status					
T1	262	179	83	14.271	0.001
T2	603	333	270		
T3–T4	164	89	75		
Lymph node status					
N0	499	291	208	4.297	0.117
N1	345	213	132		
N2–N3	185	97	88		
Metastasis status					
M0	1,013	595	418	2.924	0.087
M1	16	6	10		

### A 14 CpGs signature associated with prognosis of patients with BC

Univariate and multivariate Cox proportional hazard regression analyses for 10-year PFI indicated that higher risk scores were correlated with higher incidences of clinical events (univariate analysis, HR = 2.717, *p* < 0.001; multivariate analysis, HR = 2.498, *p* < 0.001). Additionally, univariate Cox proportional hazard regression analysis for 10-year PFI indicated that higher age, tumor size status, lymph node status, and metastasis status were correlated with higher incidences of clinical events [age (41–60 years vs. < 41 years), HR = 0.481, *p* = 0.004; tumor size status (T2 vs. T1), HR = 1.854, *p* = 0.013, (T3–T4 vs. T1), HR = 3.588, *p* < 0.001; lymph node status (N1 vs. N0), HR = 1.646, *p* = 0.018, (N2–N3 vs. T0), HR = 3.181, *p* < 0.001; metastasis status, (M1 vs. M0), HR = 7.841, *p* < 0.001]. Factors with statistical significance in the univariate analysis were further included in the multivariate analysis, which indicated that higher age (41–60 years vs. <41 years), tumor size status (T2 and T3–T4 vs. T1), lymph node status (N1 and N2–N3 vs. N0), and metastasis status (M1 vs. M0) were correlated with higher incidences of clinical events [age (41–60 years vs. <41 years), HR = 0.468, *p* = 0.003; tumor size status (T2 vs. T1), HR = 1.373, *p* = 0.218, (T3–T4 vs. T1), HR = 1.956, *p* = 0.025; lymph node status (N1 vs. N0), HR = 1.412, *p* = 0.113, (N2–N3 vs. N0), HR = 2.040, *p* < 0.006; metastasis status, (M1 vs. M0), HR = 3.632, *p* < 0.001]. The results of univariate and multivariate Cox proportional hazard regression analyses for 10-year PFI are shown in Table 3. The 14-CpG signature serves as a strong independent predictor of BC prognosis, with higher risk scores correlating with worse clinical outcomes. Additionally, established clinical factors such as tumor size, lymph node involvement, and metastasis status were validated as independent predictors of disease progression. These findings suggest that integrating the 14-CpG signature with traditional clinicopathological features could enhance risk stratification and guide personalized treatment strategies.

**Table 3:** Univariate and multivariate Cox proportional hazard models of PFI in BC.

Variables	Univariate			Multivariate		
	HR	95% CI	*p*-value	HR	95% CI	*p*-value
Age						
41–60 years	0.481	0.293–0.789	0.004	0.468	0.282–0.775	0.003
>60 years	0.704	0.432–1.147	0.159	0.638	0.384–1.061	0.083
Molecular subtype						
Luminal A	0.681	0.420–1.106	0.120			
Luminal B	0.700	0.371–1.306	0.260			
HER2	1.073	0.501–2.301	0.856			
Basal-like	1.201	0.703–2.054	0.502			
Tumor size status						
T2	1.854	1.137–3.024	0.013	1.373	0.829–2.273	0.218
T3–T4	3.588	2.099–6.133	<0.001	1.956	1.089–3.511	0.025
Lymph node status						
N1	1.646	1.090–2.486	0.018	1.412	0.921–2.387	0.113
N2–N3	3.181	2.034–4.976	<0.001	2.040	1.232–3.377	0.006
Metastasis status						
M1	7.841	4.407–13.942	<0.001	3.632	1.898–6.951	<0.001
Risk group						
High risk	2.717	1.897–3.891	<0.001	2.498	1.730–3.607	<0.001

### Evaluation of the predictive power of the prognostic signature

According to previous studies, factors such as age, intrinsic molecular subtype (PAM50), and TNM stage are closely linked to prognosis in patients with BC (26–28). To validate the potential of the prognostic signature as a predictor of progression-free interval (PFI), disease-specific survival (DSS), and overall survival (OS) in BC patients, the entire TCGA BC dataset was stratified by TNM stage, age, and molecular subtype. Patients were divided into three age subgroups (<41, 41–60, and >60 years), five molecular subgroups (PAM50: luminal A, luminal B, HER2, basal like, and normal like), and four TNM stage subgroups (I, II, III, and IV).

**Figure 2:**
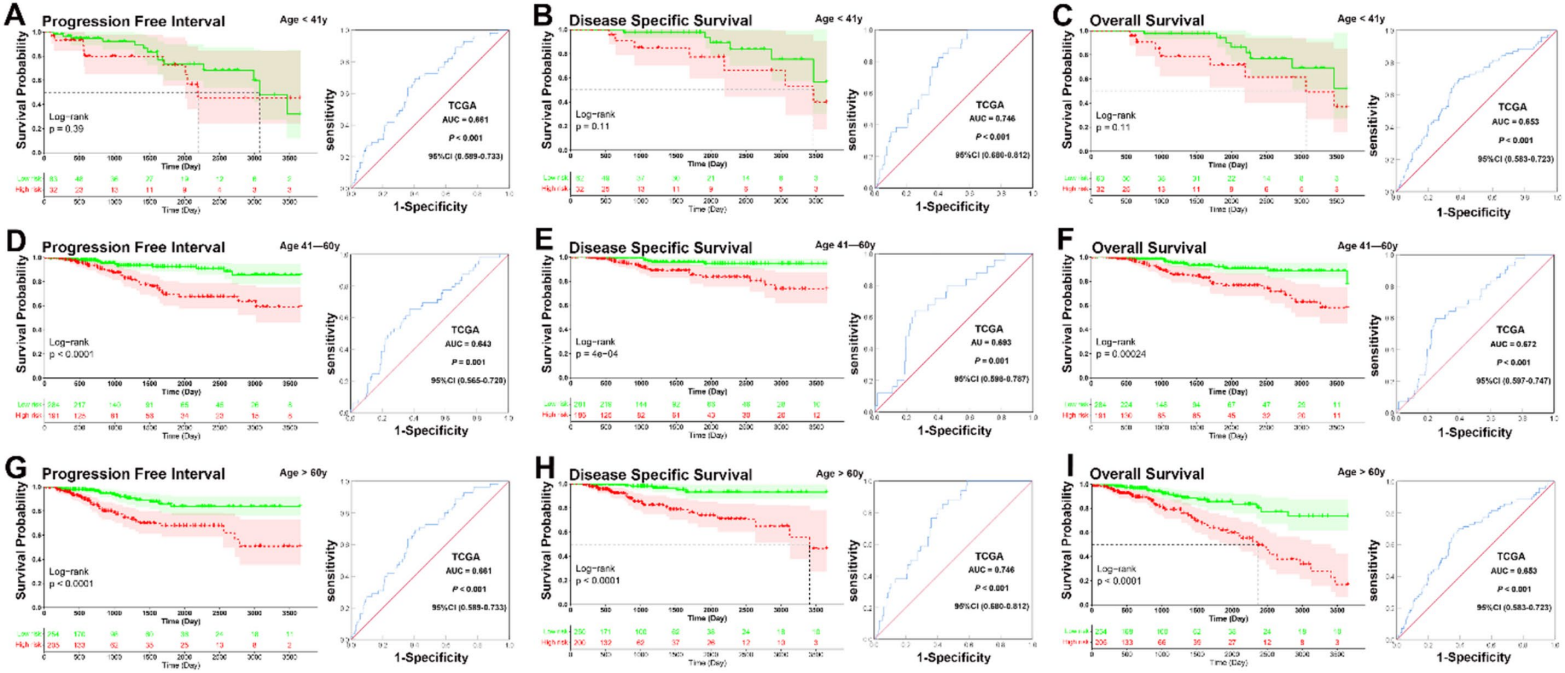
KM and ROC curve analysis of patients stratified by age. **(A–C)** KM analysis indicated that there was no no significantly longer PFI, DSS, and OS between patients in the low-risk and high-risk groups in the <41 years subgroup (<41 years, PFI, *p* = 0.39, DSS, *p* = 0.11, OS, *p* = 0.11). ROC curve analysis demonstrated that the prognostic signature had good sensitivity and specificity for predicting PFI, DSS, and OS in the <41 years subgroup (PFI, AUC = 0.661, 95% CI 0.589–0.733, *p* < 0.001, DSS, AUC = 0.746, 95% CI 0.680–0.812, *p* < 0.001, OS, AUC = 0.653, 95% CI 0.583–0.723, *p* < 0.001). **(D–F)** KM analysis indicated that patients in the low-risk group had significantly longer PFI, DSS, and OS in the 41–60 years subgroup (all *p* < 0.001). ROC curve analysis demonstrated that the prognostic signature had good sensitivity and specificity for predicting PFI, DSS, and OS in the 41–60 years subgroup (PFI, AUC = 0.643, 95% CI 0.565–0.720, *p* = 0.001, DSS, AUC = 0.693, 95% CI 0.598–0.787, *p* = 0.001, OS, AUC = 0.672, 95% CI 0.597–0.747, *p* < 0.001). **(G–I)** KM analysis indicated that patients in the low-risk group had significantly longer PFI, DSS, and OS in the >60 years subgroup (all *p* < 0.001). ROC curve analysis demonstrated that the prognostic signature had good sensitivity and specificity for predicting PFI, DSS, and OS in the >60 years subgroup (PFI, AUC = 0.661, 95% CI 0.589–0.733, *p* < 0.001, DSS, AUC = 0.746, 95% CI 0.680–0.812, *p* < 0.001, OS, AUC = 0.653, 95% CI 0.583–0.723, *p* < 0.001).

KM analysis indicated that patients in the low-risk group had significantly longer PFI, DSS, and OS in the 41–60 and >60 years subgroups (all *p* < 0.001), but not in the <41 years subgroup (<41 years, PFI, *p* = 0.39, DSS, *p* = 0.11, OS, *p* = 0.11). ROC curve analysis demonstrated that the prognostic signature had good sensitivity and specificity for predicting PFI, DSS, and OS across all three age subgroups (<41 years, PFI, AUC = 0.661, DSS, AUC = 0.746, OS, AUC = 0.653, all *p* < 0.001; 41–60 years, PFI, AUC = 0.643, *p* = 0.001, DSS, AUC = 0.693, *p* = 0.001, OS, AUC = 0.672, *p* < 0.001; >60 years, PFI, AUC = 0.661; DSS, AUC = 0.746; OS, AUC = 0.653, all *p* < 0.001) (Figures 2A–I). KM survival analysis revealed that the signature may be particularly predictive in older patients, where it provides meaningful prognostic differentiation. ROC analysis further confirmed the prognostic value of the signature across all age subgroups, showing good sensitivity and specificity for predicting PFI, DSS, and OS.

**Figure 3:**
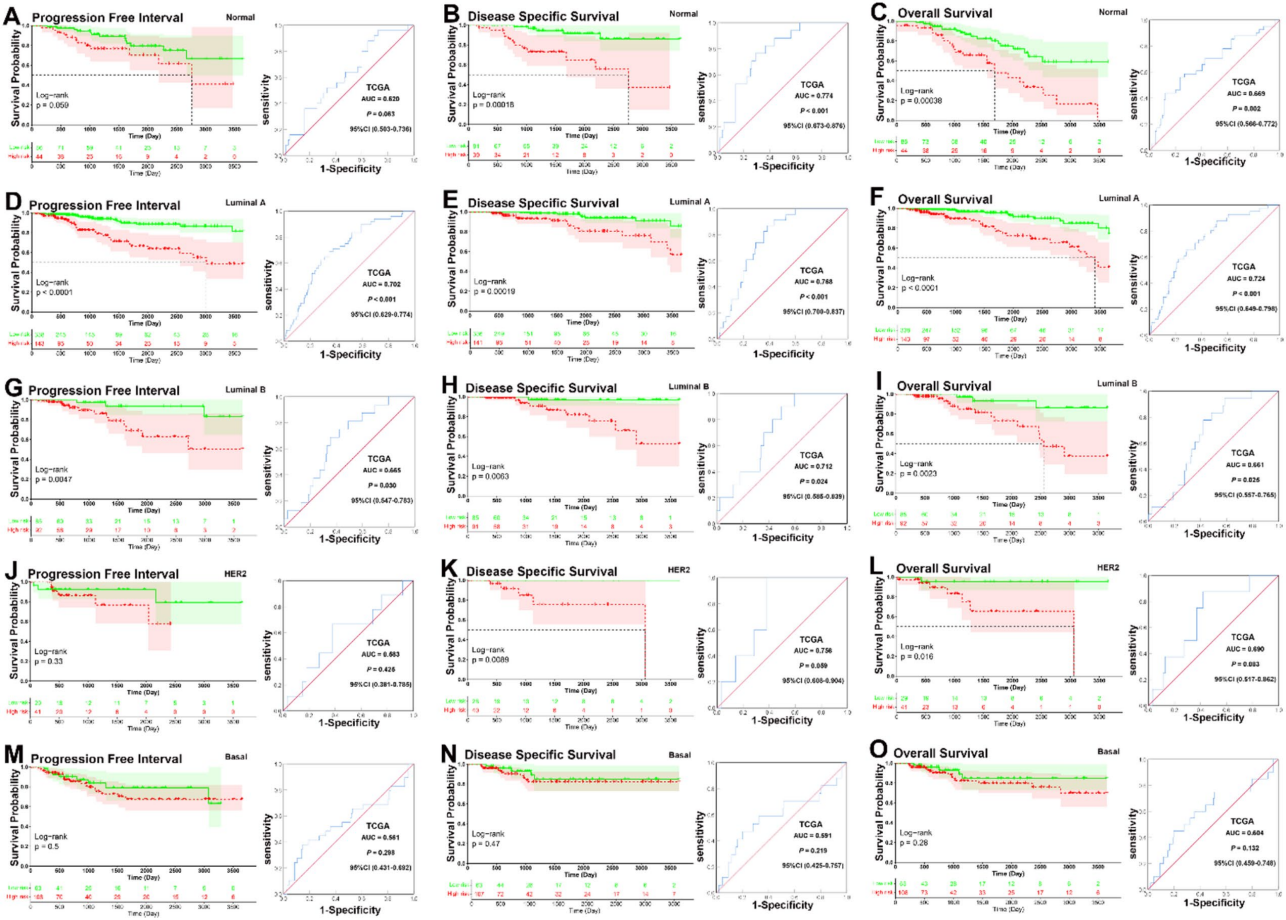
KM and ROC curve analysis of patients stratified by molecular subtype (PAM50). **(A–C)** KM curves demonstrated that patients in the low-risk group had significantly better prognoses than those in the high-risk group in the normal-like subgroup (PFI, *p* = 0.059, DSS, *p* < 0.001, OS, *p* < 0.001); ROC analysis demonstrated that the signature had good sensitivity and specificity for predicting PFI, DSS, and OS in the normal-like subgroup (PFI, AUC = 0.620, 95% CI 0.503–0.736, *p* = 0.0063, DSS, AUC = 0.774, 95% CI 0.673–0.876, *p* < 0.001, OS, AUC = 0.669, 95% CI 0.566–0.772, *p* = 0.002). **(D–F)** KM curves demonstrated that patients in the low-risk group had significantly better prognoses than those in the high-risk group in the luminal A subgroup (all *p* < 0.001); ROC analysis demonstrated that the signature had good sensitivity and specificity for predicting PFI, DSS, and OS in the luminal A subgroup (PFI, AUC = 0.702, 95% CI 0.629–0.774, *p* < 0.001, DSS, AUC = 0.768, 95% CI 0.700–0.837, *p* < 0.001, OS, AUC = 0.724, 95% CI 0.649–0.798, *p* < 0.001). **(G–I)** KM curves demonstrated that patients in the low-risk group had significantly better prognoses than those in the high-risk group in the luminal B subgroup (PFI, *p* = 0.0047, DSS, *p* = 0.0063, OS, *p* = 0.0023); ROC analysis demonstrated that the signature had good sensitivity and specificity for predicting PFI, DSS, and OS in the luminal B subgroup (PFI, AUC = 0.665, 95% CI 0.547–0.783, *p* = 0.030, DSS, AUC = 0.712, 95% CI 0.585–0.839, *p* = 0.024, OS, AUC = 0.661, 95% CI 0.557–0.765, *p* = 0.025). **(J–L)** KM curves demonstrated that patients in the low-risk group had significantly better prognoses than those in the high-risk group in the HER2 subgroup (PFI, *p* = 0.33, DSS, *p* = 0.0089, OS, *p* = 0.016); ROC analysis showed that there was no significantly better sensitivity and specificity for predicting PFI, DSS, and OS in the HER2 subgroup (PFI, AUC = 0.583, 95% CI 0.381–0.785, *p* = 0.425; DSS, AUC = 0.756, 95% CI 0.608–0.904, *p* = 0.059; OS, AUC = 0.690, 95% CI 0.517–0.862, *p* = 0.083). **(M–O)** KM analysis indicated that there was no significantly longer PFI, DSS, and OS between patients in the low-risk and high-risk groups in the basal-like subgroup (PFI, *p* = 0.5, DSS, *p* = 0.47, OS, *p* = 0.28); ROC analysis showed that there was no significantly better sensitivity and specificity for predicting PFI, DSS, and OS in the basal-like subgroup (PFI, AUC = 0.561, 95% CI 0.431–0.692, *p* = 0.298, DSS, AUC = 0.591, 95% CI 0.425–0.757, *p* = 0.219, OS, AUC = 0.604, 95% CI 0.459–0.748, *p* = 0.132).

In the analysis of the five molecular subtype subgroups, overall KM curves also demonstrated that patients in the low-risk group had significantly better prognoses than those in the high-risk group in the normal-like, luminal A, luminal B, and HER2 subgroups [normal-like, PFI, *p* = 0.059, DSS, *p* < 0.001, OS, *p* < 0.001 (Figures 3A–C); luminal A, all *p* < 0.001 (Figures 3D–F); luminal B, PFI, *p* = 0.0047, DSS, *p* = 0.0063, OS, *p* = 0.0023 (Figures 3G–I); HER2, PFI, *p* = 0.33, DSS, *p* = 0.0089, OS, *p* = 0.016 (Figures 3J–L)], but not basal like subgroup [basal-like, PFI, *p* = 0.5, DSS, *p* = 0.47, OS, *p* = 0.28 (Figures 3M–O)]. Except for the HER2 and basal-like subgroups, ROC analysis also demonstrated that the signature had good sensitivity and specificity for predicting PFI, DSS, and OS in the other molecular subtype subgroups [normal-like, PFI, AUC = 0.620, *p* = 0.0063, DSS, AUC = 0.774, *p* < 0.001, OS, AUC = 0.669, *p* = 0.002 (Figures 3A–C); luminal A, PFI, AUC = 0.702, DSS, AUC = 0.768, OS, AUC = 0.724, all *p* < 0.001 (Figures 3D–F); luminal B, PFI, AUC = 0.665, *p* = 0.030, DSS, AUC = 0.712, *p* = 0.024, OS, AUC = 0.661, *p* = 0.025 (Figures 3G–I)], but not for HER2 and basal-like subgroups [HER2, PFI, AUC = 0.583, *p* = 0.425; DSS, AUC = 0.756, *p* = 0.059; OS, AUC = 0.690, *p* = 0.083 (Figures 3J–L); basal-like, PFI, AUC = 0.561, *p* = 0.298, DSS, AUC = 0.591, *p* = 0.219, OS, AUC = 0.604, *p* = 0.132 (Figures 3M–O)]. Overall, KM survival curves indicated that patients in the low-risk group had significantly better prognoses in the normal-like, luminal A, luminal B, and HER2 subgroups, except in the basal-like subgroup, where no significant differences were observed. ROC analysis confirmed that the signature was predictive in the normal-like, luminal A, and luminal B subgroups, while it performed less well in the HER2 and basal-like subgroups.

**Figure 4:**
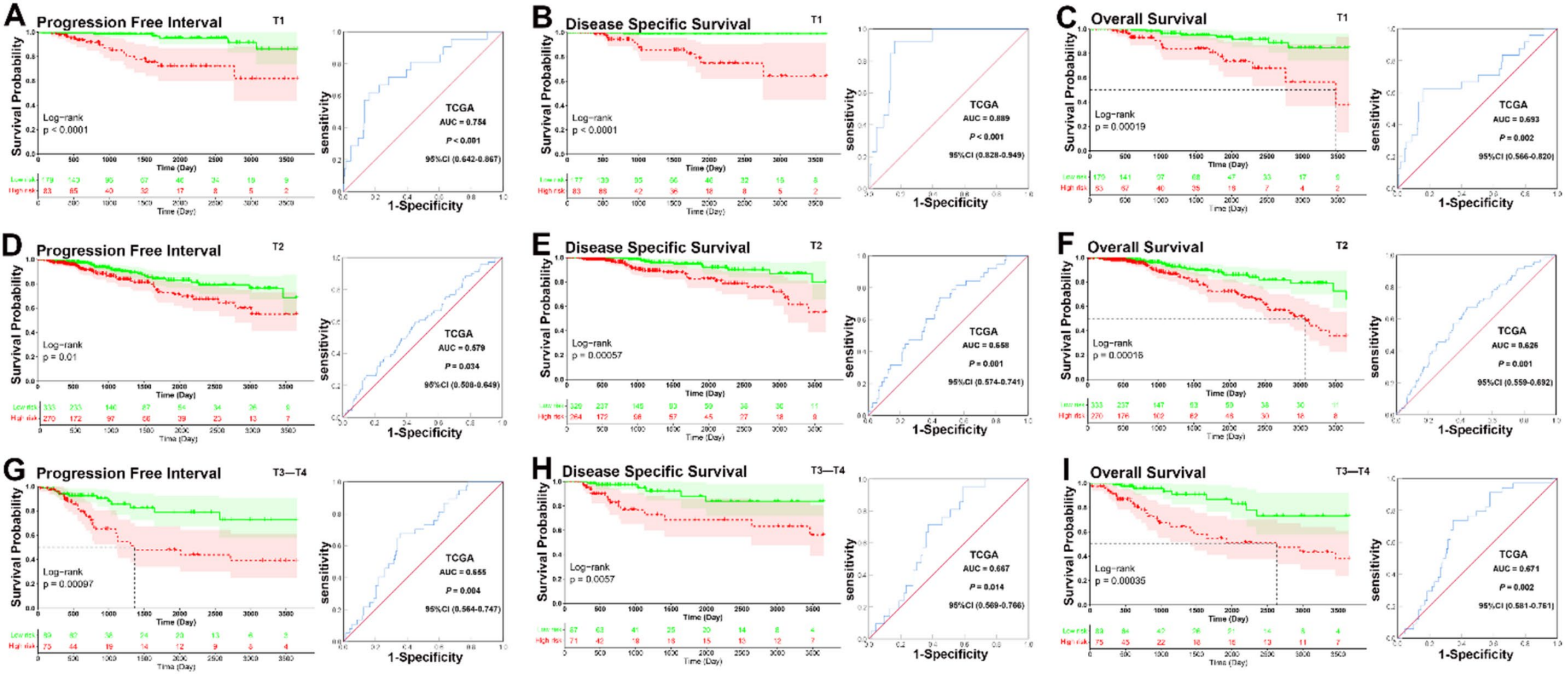
KM and ROC curve analyses of patients stratified by tumor size status. **(A–C)** KM curves demonstrated that patients in the low-risk group had significantly better prognoses than those in the high-risk group in T1 subgroup (PFI, *p* < 0.001, DSS, *p* < 0.001, OS, *p* < 0.001); ROC analysis demonstrated that the signature had good sensitivity and specificity for predicting PFI, DSS, and OS in T1 subgroup (PFI, AUC = 0.754, 95% CI 0.642–0.867, *p* < 0.001; DSS, AUC = 0.889, 95% CI 0.828–0.949, *p* < 0.001; OS, AUC = 0.693, 95% CI 0.566–0.820, *p* = 0.002). **(D–F)** KM curves demonstrated that patients in the low-risk group had significantly better prognoses than those in the high-risk group in T2 subgroup (PFI, *p* = 0.01, DSS, *p* < 0.001, OS, *p* < 0.001); ROC analysis demonstrated that the signature had good sensitivity and specificity for predicting PFI, DSS, and OS in T2 subgroup (PFI, AUC = 0.579, 95% CI 0.508–0.649, *p* = 0.034; DSS, AUC = 0.658, 95% CI 0.574–0.741, *p* = 0.001; OS, AUC = 0.626, 95% CI 0.559–0.692, *p* = 0.001). **(G–I)** KM curves demonstrated that patients in the low-risk group had significantly better prognoses than those in the high-risk group in T3–T4 subgroup (PFI, *p* < 0.001, DSS, *p* = 0.0057, OS, *p* < 0.001); ROC analysis demonstrated that the signature had good sensitivity and specificity for predicting PFI, DSS, and OS in T3–T4 subgroup (PFI, AUC = 0.655, 95% CI 0.564–0.747, *p* = 0.004; DSS, AUC = 0.667, 95% CI 0.569–0.766, *p* < 0.014; OS, AUC = 0.671, 95% CI 0.581–0.761, *p* = 0.002).

In analyses of tumor size subgroups, KM curves also showed that patients in the low-risk group had a significantly better prognosis for PFI, DSS, and OS than those in the high-risk group (T1, PFI, DSS, and OS, all *p* < 0.001; T2, PFI, *p* = 0.01, DSS, *p* < 0.001, OS, *p* < 0.001; T3–T4, PFI, *p* < 0.001, DSS, *p* = 0.0057, OS, *p* < 0.001) (Figures 4A–I). ROC analysis demonstrated that the signature had good sensitivity and specificity for predicting PFI, DSS, and OS in all three tumor size status subgroups (T1, PFI, AUC = 0.754, *p* < 0.001; DSS, AUC = 0.889, *p* < 0.001; OS, AUC = 0.693, *p* = 0.002; T2, PFI, AUC = 0.579, *p* = 0.034; DSS, AUC = 0.658, *p* = 0.001; OS, AUC = 0.626, *p* = 0.001; T3–T4, PFI, AUC = 0.655, *p* = 0.004; DSS, AUC = 0.667, *p* < 0.014; OS, AUC = 0.671, *p* = 0.002) (Figures 4A–I). The KM curves and ROC analysis of tumor size subgroups demonstrated that the prognostic signature was predictive across all tumor stages.

**Figure 5:**
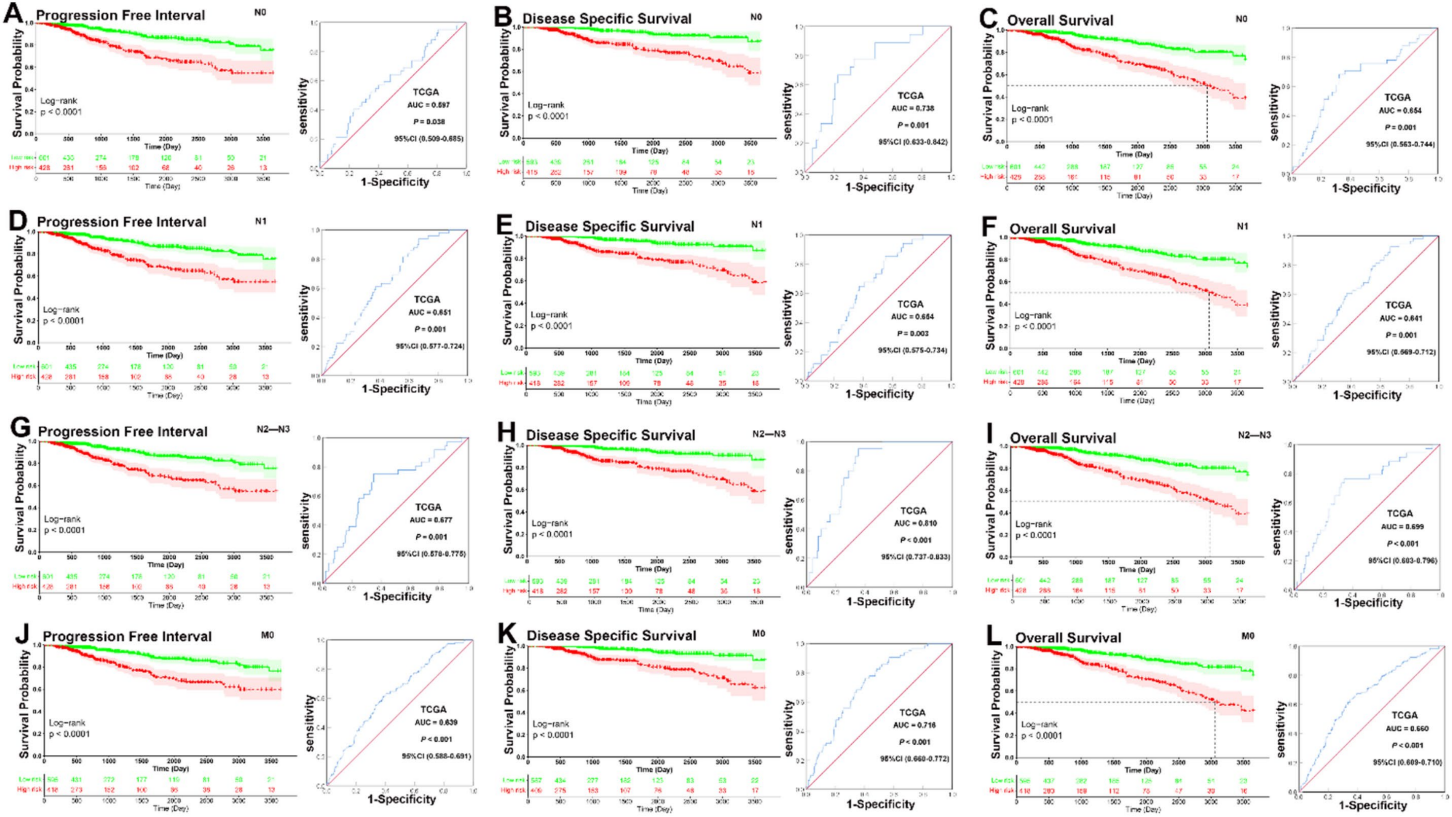
KM and ROC curve analyses of patients stratified by lymph node and metastasis status. **(A–C)** KM curves demonstrated that patients in the low-risk group had significantly better prognoses than those in the high-risk group in N0 subgroup (PFI, *p* < 0.001, DSS, *p* < 0.001, OS, *p* < 0.001); ROC analysis demonstrated that the signature had good sensitivity and specificity for predicting PFI, DSS, and OS in N0 subgroup (PFI, AUC = 0.597, 95% CI 0.509–0.685, *p* = 0.038, DSS, AUC = 0.738, 95% CI 0.633–0.842, *p* = 0.001, OS, AUC = 0.654, 95% CI 0.563–0.744, *p* = 0.001). **(D–F)** KM curves demonstrated that patients in the low-risk group had significantly better prognoses than those in the high-risk group in N1 subgroup (PFI, *p* < 0.001, DSS, *p* < 0.001, OS, *p* < 0.001); ROC analysis demonstrated that the signature had good sensitivity and specificity for predicting PFI, DSS, and OS in N0 subgroup (PFI, AUC = 0.651, 95% CI 0.577–0.724, *p* = 0.001, DSS, AUC = 0.654, 95% CI 0.575–0.734, *p* = 0.003, OS, AUC = 0.641, 95% CI 0.569–0.712, *p* = 0.001). **(G–I)** KM curves demonstrated that patients in the low-risk group had significantly better prognoses than those in the high-risk group in N1 subgroup (PFI, *p* < 0.001, DSS, *p* < 0.001, OS, *p* < 0.001); ROC analysis demonstrated that the signature had good sensitivity and specificity for predicting PFI, DSS, and OS in N0 subgroup (PFI, AUC = 0.677, 95% CI 0.578–0.775, *p* = 0.001, DSS, AUC = 0.810, 95% CI 0.737–0.833, *p* < 0.001, OS, AUC = 0.699, 95% CI 0.603–0.796, *p* < 0.001). **(J–L)** KM curves demonstrated that patients in the low-risk group had significantly better prognoses than those in the high-risk group in M0 subgroup (PFI, *p* < 0.001, DSS, *p* < 0.001, OS, *p* < 0.001); ROC analysis demonstrated that the signature had good sensitivity and specificity for predicting PFI, DSS, and OS in M0 subgroup (PFI, AUC = 0.639, 95% CI 0.588–0.691, *p* < 0.001; DSS, AUC = 0.716, 95% CI 0.660–0.772, *p* < 0.001; OS, AUC = 0.660, 95% CI 0.609–0.710, *p* < 0.001).

In KM analyses, the curves showed that patients in the low-risk group had a significantly better prognosis for PFI, DSS, and OS than those in the high-risk group across all lymph node subgroups (PFI, all the N0, N1, and N2–N3 subgroup, *p* < 0.001; DSS, all the N0, N1, and N2–N3 subgroup, *p* < 0.001; OS, all the N0, N1 and N2–N3 subgroup, *p* < 0.001). ROC analysis demonstrated that the signature had good sensitivity and specificity for predicting PFI, DSS, and OS in all three lymph node status subgroups (N0 subgroup, PFI, AUC = 0.597, *p* = 0.038, DSS, AUC = 0.738, *p* = 0.001, OS, AUC = 0.654, *p* = 0.001; N1 subgroup, PFI, AUC = 0.651, *p* = 0.001, DSS, AUC = 0.654, *p* = 0.003, OS, AUC = 0.641, *p* = 0.001; N2–N3 subgroup, PFI, AUC = 0.677, *p* = 0.001, DSS, AUC = 0.810, *p* < 0.001, OS, AUC = 0.699, *p* < 0.001) (Figures 5A–I).

In analyses of metastasis status subgroups, KM curves also showed that patients in the low-risk group had a significantly better prognosis for PFI, DSS, and OS than those in the high-risk group (all *p* < 0.0001). ROC analysis demonstrated that the signature had good sensitivity and specificity for predicting PFI, DSS, and OS in M0 subgroup (PFI, AUC = 0.639, *p* < 0.001; DSS, AUC = 0.716, *p* < 0.001; OS, AUC = 0.660, *p* < 0.001) (Figures 5J–L). Overall, these analyses indicate that the prognostic signature has a strong predictive value. Lymph node status also showed that the prognostic signature could significantly predict survival outcomes across N0, N1, and N2–N3 subgroups. The ROC analysis demonstrated that the signature had good predictive power for all lymph node status subgroups. Metastasis status analysis confirmed that the low-risk group had a significantly better prognosis for PFI, DSS, and OS compared to the high-risk group in both M0 and M1 patients. For M0 patients, the ROC analysis showed good predictive performance.

Overall, these analyses highlight the strong predictive power of the 14-CpG signature across different clinical subgroups, further validating its potential as a robust tool for personalized prognosis in BC patients.

### Nomogram development

**Figure 6:**
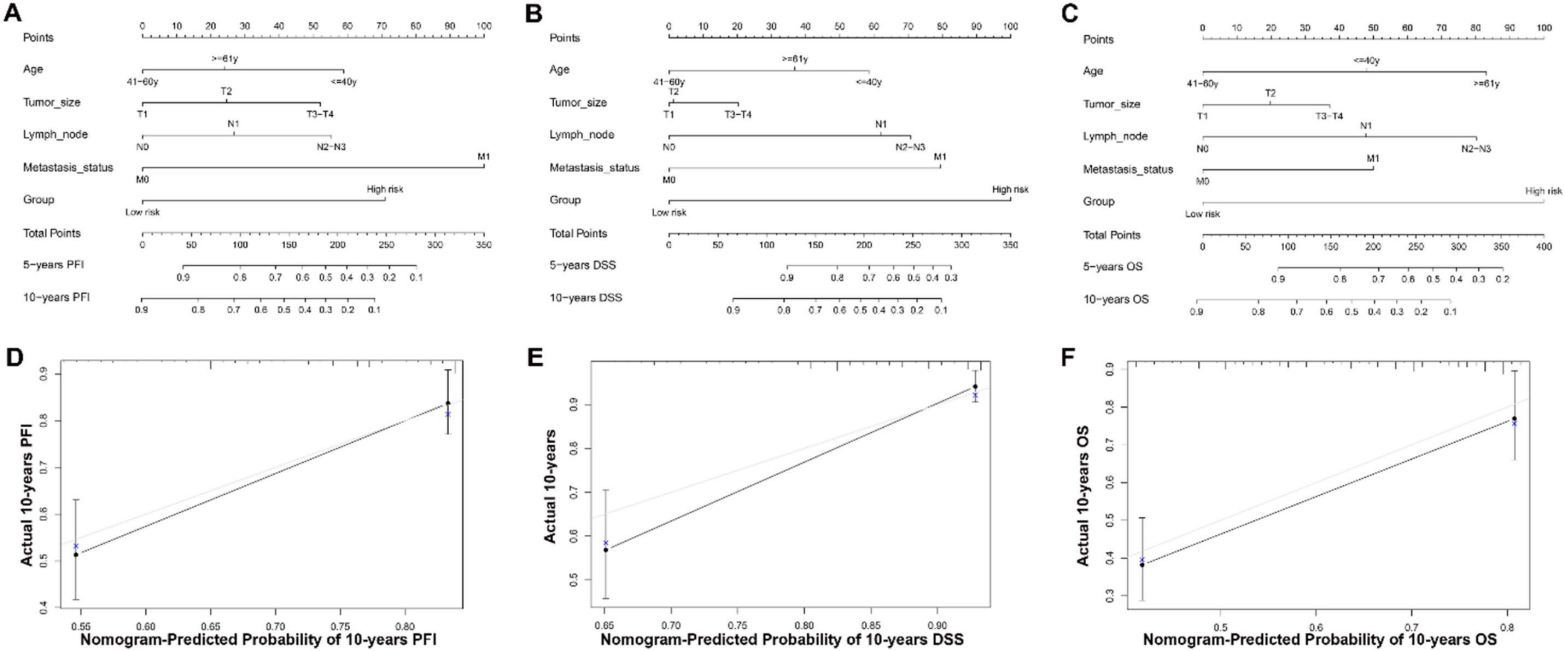
Nomogram for predicting 5- and 10-year PFI, DSS and OS of patients with BC and calibration curves of the nomogram. **(A–C)** A nomogram incorporating age, TNM status, and risk group was a predictor for 5- and 10-year PFI, DSS and OS. The sum of these points represents the total risk score, which can then be used to determine the probability of 5- and 10-year PFI, DSS, or OS by reading straight down to the corresponding axis. **(D–F)** Calibrate plots were applied for investigating the deviation in nomogram-predicted of 5- and 10-year PFI, DSS and OS. Calibration curves show that the actual probability corresponded closely to the prediction of nomogram.

Based on the results of univariate and multivariate Cox proportional hazard regression analysis, an individualized nomogram signature was developed, incorporating clinical factors (age and TNM status) along with the prognostic signature. Each risk factor corresponds to a designated point, determined by drawing a line perpendicular to the point’s axis. The sum of these points represents the total risk score, which can then be used to determine the probability of 5- and 10-year PFI, DSS, or OS by reading straight down to the corresponding axis (Figures 6A–C). The calibration curves, generated after 1,000 bootstraps, demonstrated high consistency between the nomogram-predicted probabilities and the actual probabilities for 5- and 10-year PFI, DSS, and OS in BC patients (Figures 6D–F). Our data suggest that the nomograms for PFI, DSS, and OS exhibit strong predictive efficacy for the 5- and 10-year probabilities. This nomogram serves as a valuable tool for clinicians to estimate patient prognosis more accurately, integrating both molecular and clinical data.

### Inspiration for chemotherapy decision-making

**Figure 7:**
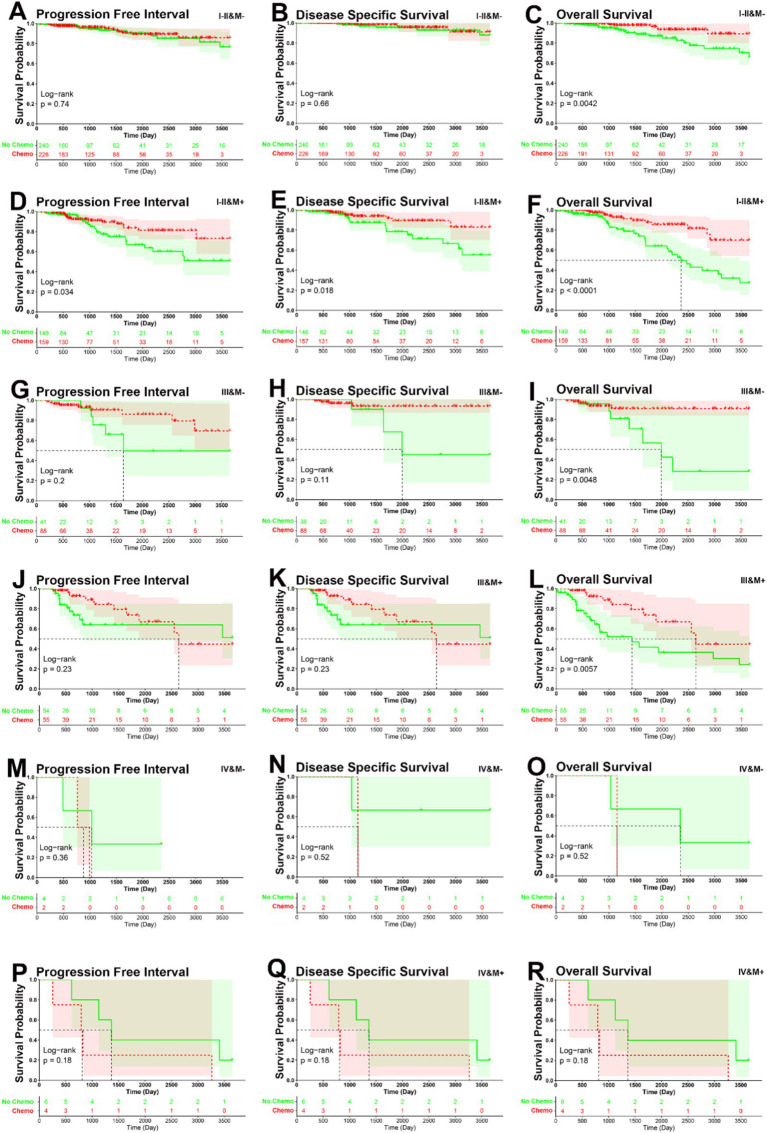
KM curve analysis of patients stratified by six risk subgroups. **(A–C)** KM curves demonstrated that patients who underwent adjuvant chemotherapy had significantly better OS than those who did not, but not for PFI and DSS in I–II & M^−^ subgroup (PFI, *p* = 0.74, DSS, *p* = 0.66, OS, *p* = 0.0042). **(D–F)** KM curves demonstrated that patients who underwent adjuvant chemotherapy had significantly better PFI, DSS, and OS than those who did not in I–II & M^+^ subgroup (PFI, *p* = 0.034, DSS, *p* = 0.018, OS, *p* < 0.001). **(G–I)** KM curves demonstrated that patients who underwent adjuvant chemotherapy had significantly better OS than those who did not, but not for PFI and DSS in III & M^−^ subgroup (PFI, *p* = 0.2, DSS, *p* = 0.11, OS, *p* = 0.0048). **(J–L)** KM curves demonstrated that patients who underwent adjuvant chemotherapy had significantly better OS than those who did not, but not for PFI and DSS in III & M^+^ subgroup (PFI, *p* = 0.4, DSS, *p* = 0.23, OS, *p* = 0.0057). **(M–O)** KM curves demonstrated that patients who underwent adjuvant chemotherapy had no significantly better PFI, DSS, and OS than those who did not in IV & M^−^ subgroup (PFI, *p* = 0.36, DSS, *p* = 0.36, OS, *p* = 0.52). **(P–R)** KM curves demonstrated that patients who underwent adjuvant chemotherapy had no significantly better PFI, DSS, and OS than those who did not in IV & M^+^ subgroup (PFI, *p* = 0.32, DSS, *p* = 0.18, OS, *p* = 0.18).

Combining the TNM stage and DNA methylation risk group (low-risk group labeled as M^−^, and high-risk labeled as M^+^), the total patients were classified into the following six subgroups: I–II & M^−^ (*N* = 466), I–II & M^+^ (*N* = 308), III & M^−^ (*N* = 129), III & M^+^ (*N* = 110), IV & M^−^ (*N* = 6), and IV & M^+^ (*N* = 10). In the I–II & M^+^ subgroup, patients who underwent adjuvant chemotherapy had significantly better PFI, DSS, and OS than those who did not (PFI, *p* = 0.034, DSS, *p* = 0.018, OS, *p* < 0.001). In the I–II & M^−^, III & M^−^, and III & M^+^ subgroups, patients who underwent adjuvant chemotherapy had significantly better OS than those who did not (I–II & M^−^, *p* = 0.0042, III & M^−^, *p* = 0.0048, III & M^+^, *p* = 0.0057), but no significant between-group difference was observed in PFI and DSS (I–II & M^−^, PFI, *p* = 0.74, DSS, *p* = 0.66; III & M^−^, PFI, *p* = 0.2, DSS, *p* = 0.11; III & M^+^, PFI, *p* = 0.4, DSS, *p* = 0.23). However, patients did not obtain significant benefit from adjuvant chemotherapy in the IV & M^−^ and IV & M^+^ subgroups (IV & M^−^, PFI, *p* = 0.36, DSS, *p* = 0.52, OS, *p* = 0.52, IV & M^+^, PFI, *p* = 0.32, DSS, *p* = 0.18, OS, *p* = 0.18) (Figures 7A–R). This analysis highlights that adjuvant chemotherapy decisions could be refined by considering both TNM stage and methylation risk group. Early-stage high-risk (I–II & M^+^) patients are likely to benefit the most from chemotherapy, advanced-stage (IV) patients, regardless of methylation risk, may require alternative therapeutic strategies beyond standard chemotherapy.

### Bioinformatic and drug sensitivity analysis of the two prognosis risk groups revealed significant findings

**Figure 8:**
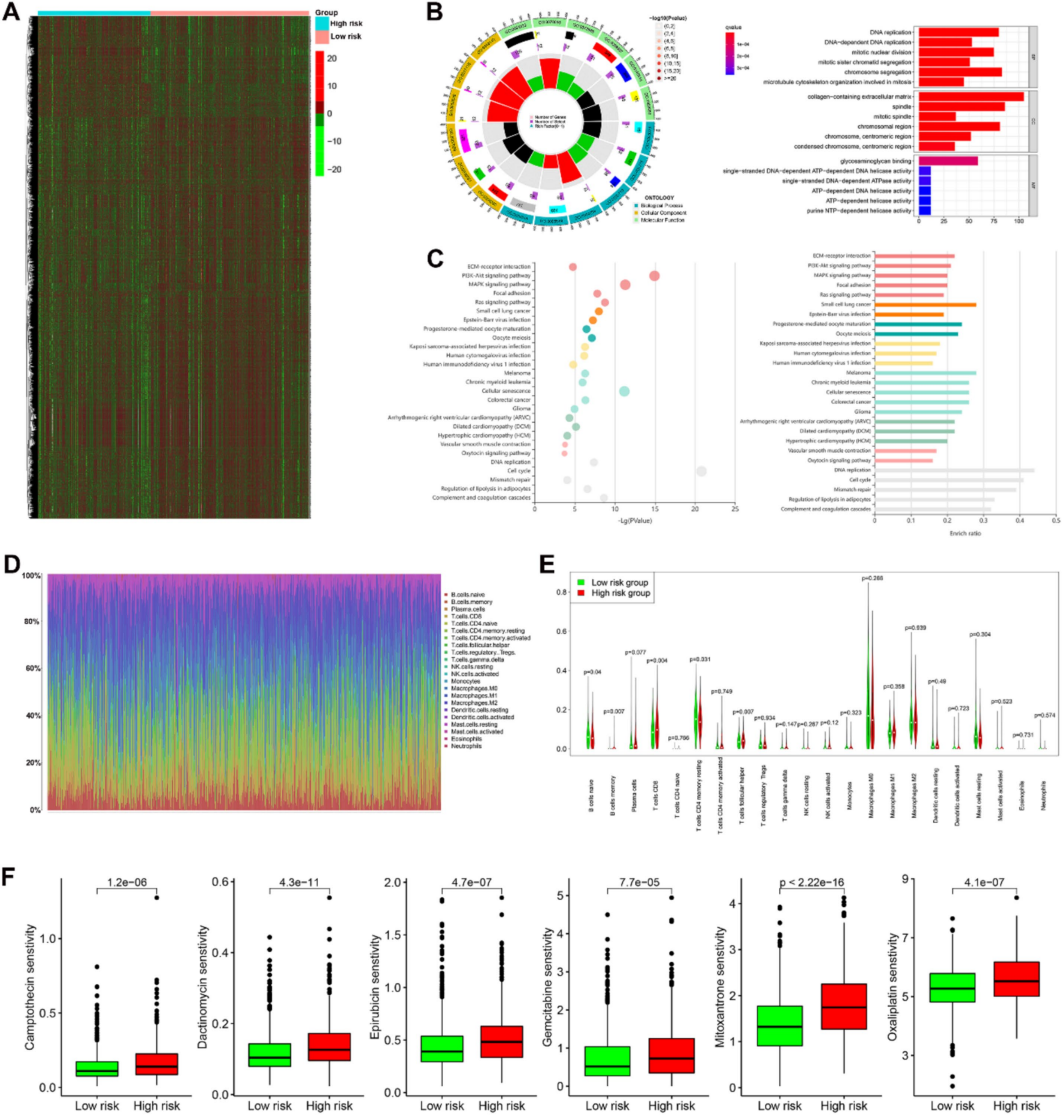
Biological functions and interactions of the differential gene expressions (DGEs) in the two prognosis risk groups in BC. **(A)** A total of 7,537 differentially expressed genes between the two prognosis risk groups was shown in heatmap. **(B,C)** Gene Ontology (GO) annotation [biological process (BP), cellular component (CC), and molecular function (MF)] and KEGG pathway analysis analyses indicated enrichment of the top 3,000 DEGs in pathways related to DNA replication and repair, as well as cell cycle regulation. **(D)** Distinct landscapes of 22 types of immunocyte infiltration between the two prognosis risk groups. **(E)** Significant differences were observed in the distribution of certain immune cell types, including B cells naive, B cells memory, T cells CD8, T cells CD4 memory resting, and T cells follicular helper in the two prognostic risk groups. **(F)** Significant differences in sensitivity of some drugs (camptothecin, dactinomycin, epirubicin, gemcitabine, mitoxantrone, and oxaliplatin) in the two prognostic risk groups.

Differential gene expression analysis using the Wilcoxon test identified a total of 7,537 differentially expressed genes (DEGs) with FDR ≤0.01, as shown in the expression heatmap (Figure 8A). Subsequent GO functional enrichment (Figure 8B) and KEGG pathway (Figure 8C) analyses indicated enrichment of the top 3,000 DEGs in pathways related to DNA replication and repair, as well as cell cycle regulation. Examples include DNA replication, DNA-dependent DNA replication, mitotic nuclear division, mitotic sister chromatid segregation, chromosome segregation, spindle, mitotic region, and single-stranded DNA-dependent ATP-dependent DNA helicase activity. Additionally, pathways such as ECM-receptor interaction, PI3K-Akt signaling pathway, MAPK signaling pathway, and cellular senescence were highlighted. To assess the tumor microenvironment, CIBERSORT analysis was performed, revealing distinct landscapes of tumor-infiltrating immune cells between the two prognosis risk groups, as depicted in the barplot (Figure 8D). Significant differences were observed in the distribution of certain immune cell types, including B cells naive, B cells memory, T cells CD8, T cells CD4 memory resting, and T cells follicular helper (Figure 8E). Utilizing the GDSC database, significant differences in sensitivity to chemotherapy drugs and immunomodulating drugs were identified between the high and low risk groups. Examples include camptothecin, dactinomycin, epirubicin, gemcitabine, mitoxantrone, and oxaliplatin (Figure 8F). The distinct molecular pathways and immune landscapes between risk groups may inform targeted therapy decisions. High-risk patients may benefit from chemotherapy and immune-modulating agents, as indicated by their unique drug sensitivity profiles. This analysis supports the prognostic signature’s potential in guiding personalized treatment strategies beyond traditional clinicopathological factors.

## Discussion

Breast cancer had the highest incidence and one of the highest mortality rates among cancers threatening the health of females worldwide in 2020. Therefore, it is imperative to explore novel therapeutic strategies for this deadly disease (1). Despite advancements in medical technology and the development of multigene prognostic tools like Oncotype DX and MammaPrint to aid in clinical decision-making for BC patients, these biomarkers are only applicable to a subset of patients (7, 29, 30). Due to the complexity and heterogeneity of BC, some patients do not benefit from chemotherapy, hormonal therapy, radiotherapy, and more recently, immunotherapy or targeted therapy (31, 32). The role of DNA methylation in BC is becoming clearer, and DNA methylation-based models may complement existing treatment strategies (33, 34). Therefore, constructing DNA methylation-based prognostic and diagnostic models for BC is critically needed.

In our current study, we identified a total of 216 differentially methylated CpG sites between normal and tumor samples by analyzing the TCGA, GSE22249, and GSE66695 databases (Figures 1A–D). Subsequently, univariate Cox proportional hazard analysis identified 16 CpG sites with prognostic value for BC (Figure 1E). Finally, LASSO Cox regression analysis determined that 14 of these 16 prognosis-related CpG sites constitute the optimal prognostic model for predicting PFI risk in BC patients within the TCGA dataset (Figures 1F–G). The 14 CpG sites included cg02409351, cg03943081, cg04590978, cg06516124, cg06539804, cg08348496, cg11279021, cg12880658, cg14011639, cg19591881, cg20950011, cg23917399, cg26282384, and cg26524263. Two sites (cg04590978 and cg19591881) demonstrated a protective effect, while 12 sites (cg02409351, cg03943081, cg06516124, cg06539804, cg08348496, cg11279021, cg12880658, cg14011639, cg20950011, cg23917399, cg26282384, and cg26524263) were associated with increased risk (Figure 1E).

Among these CpG sites, some were associated with malignant progression of tumors and chemotherapy resistance, while others were linked to neurological diseases. For instance, the methylation level of homeobox transcription factor ALX1, influenced by the CpG site cg02409351, can induce Snail expression to promote epithelial-to-mesenchymal transition and invasion of ovarian cancer cells (35). Additionally, the CpG site cg03943081 affects the methylation of the TCERG1L gene, with hypermethylation of TCERG1L being a potential biomarker for early detection of colorectal cancer (36). The CpG site cg04590978 influences the methylation of the CCNJ gene, which is associated with prognosis in hepatocellular carcinoma (37). Hypomethylation of the CpG site cg06516124 can increase the expression level of WT1 and is significantly associated with increased gastric cancer risk (38). The CpG site cg06539804 affects the methylation of the CPXM1 gene, which is upregulated in gastric cancer and correlated with poor prognosis (39). The CpG site cg08348496 affects the methylation of the HAPLN3 gene, which is involved in general metabolism in triple-negative BC in a homogeneous population from northeastern Mexico (40). The CpG site cg11279021 influences the methylation of the ETV1 gene, which may play significant roles in colorectal cancer development and is significantly associated with the infiltration of cancer-associated fibroblasts and M2 macrophages (41). The CpG site cg12880658 affects the methylation of the CDO1 gene, and increased CDO1 expression can suppress cell proliferation, migration, and invasion in BC cells, exerting a tumor suppressor effect by inhibiting the cell cycle, promoting apoptosis, and ferroptosis (42). The CpG sites cg14011639 and cg26282384 influence the methylation of the protocadherin gene family clusters (PCDHG), which are related to BC and meningioma (43, 44). The CpG site cg19591881 affects the methylation of the CD34 gene, with stromal loss of CD34+ fibroblasts significantly associated with lower overall and disease-free survival rates in BC (45). The CpG site cg20950011 influences the methylation of the CIDEA gene, which is involved in adipose tissue loss in cancer cachexia (46). The CpG site cg26524263 affects the methylation of the TNFAIP8 gene, with the knockdown of TNFAIP8 suppressing cell proliferation, migration, and invasion, and inducing cell cycle arrest in MDA-MB-231 cells (47). Lastly, the CpG site cg26524263 also influences the methylation of the KLK13 gene, with decreased KLK13 expression correlating with poor survival in esophageal squamous cell carcinoma (48).

Further Kaplan–Meier, ROC analyses, and univariate and multivariate Cox regression analyses demonstrated the utility of the 14-CpG-related signature as a powerful predictor of prognosis in BC patients within the TCGA dataset (Figures 1–5 and Table 3). A nomogram constructed by combining the 14-CpG-related signature and conventional prognostic factors exhibited high predictive efficacy for 5- and 10-year PFI, DSS, and OS in BC patients (Figure 6). However, further intensive analyses are required to verify the clinical application and promotion value of the signature. By combining clinical risk groups and gene risk groups, patients were classified into six subgroups: I–II & M^−^ (*N* = 466), I–II & M^+^ (*N* = 308), III & M^−^ (*N* = 129), III & M^+^ (*N* = 110), IV & M^−^ (*N* = 6), and IV & M^+^ (*N* = 10). KM analyses suggested that patients in the I–II & M^+^ subgroup could benefit from adjuvant chemotherapy for PFI, DSS, and OS. Patients in the I–II & M^−^, III & M^−^, and III & M^+^ subgroups could benefit from adjuvant chemotherapy for OS, but not for PFI and DSS. For the III&M- subgroup, patients who underwent adjuvant chemotherapy showed no statistically significant difference in PFI and DSS (PFI, *p* = 0.2; DSS, *p* = 0.11), although there was relative clinical survival benefit (Figures 7G,H). Additionally, patients in the IV & M^−^ and IV & M^+^ subgroups did not obtain significant benefits from adjuvant chemotherapy (IV & M^−^, PFI, *p* = 0.36; DSS, *p* = 0.52; OS, *p* = 0.52; IV & M^+^, PFI, *p* = 0.32; DSS, *p* = 0.18; OS, *p* = 0.18) (Figures 7M–R). Although patients in the IV & M^−^ and IV & M^+^ subgroups had no significant survival benefit from adjuvant chemotherapy, it might improve their quality of life. These results provide theoretical evidence for future clinical decision-making, and further studies are needed to validate these findings.

Cancer is characterized by uncontrolled tumor cell proliferation, aberrant cell cycle progression, and abnormal infiltration of immune cells (49). In this study, GO functional enrichment (Figure 8B) and KEGG pathway (Figure 8C) analysis revealed that the top 3,000 DGEs were enriched in pathways related to DNA replication, repair, and cell cycle regulation. These pathways include DNA replication, DNA-dependent DNA replication, mitotic nuclear division, mitotic sister chromatid segregation, chromosome segregation, spindle formation, mitotic region activity, single-stranded DNA-dependent ATP-dependent DNA helicase activity, PI3K-Akt signaling pathway, MAPK signaling pathway, and cellular senescence (Figures 8B,C). Additionally, we found statistically significant differences in the distribution of immune cells, including naive B cells, memory B cells, CD8^+^ T cells, resting memory CD4^+^ T cells, and follicular helper T cells, between the two prognostic risk groups (Figure 8E). This suggests that immune cell infiltration patterns may play a crucial role in cancer prognosis.

The GDSC database was utilized to identify potential therapeutic drugs for the prognostic risk groups. Significant differences in sensitivity to various chemotherapy and immunomodulating drugs were observed between the high-risk and low-risk groups. Notably, drugs such as camptothecin, dactinomycin, epirubicin, gemcitabine, mitoxantrone, and oxaliplatin demonstrated varying degrees of effectiveness (Figure 8F). These drugs are known to interfere with DNA replication and repair processes in tumor cells, thereby inhibiting their proliferation and growth. These findings are consistent with our bioinformatic analysis, which highlighted the importance of DNA replication and repair pathways in cancer progression.

Recent studies on BC have developed several multi-DNA methylation signatures for predicting prognosis (16–18, 50, 51). These include a 7-DNA methylation signature, a 6-gene prognostic signature based on differential DNA methylation, a 5-DNA methylation signature, and a 13-gene DNA methylation signature, among others. However, our model has several advantages as a predictor of prognosis in patients with BC. Thirdly, our results were consistent across both the training set (TCGA) and the validation sets (GSE22249 and GSE66695), indicating a high degree of confidence in our findings. Additionally, our model suggests that patients in the I-II&M+ subgroup could benefit from adjuvant chemotherapy for PFI, DSS, and OS. Patients in the I–II & M^−^, III & M^−^, and III & M^+^ subgroups could benefit from adjuvant chemotherapy for OS, but not for PFI and DSS. These insights can inform clinical decision-making regarding appropriate treatment strategies for BC patients. Furthermore, GO functional enrichment and KEGG pathway analysis suggests that high-risk patients might benefit from drugs that interfere with the DNA replication and repair processes of tumor cells.

Despite the strengths of our study, several limitations and challenges must be addressed. First, translating these findings into real-world clinical settings may take time, and while limited by follow-up duration and sample size, our Breast Center has already initiated the establishment of a validation dataset to confirm these results. If validated, we plan to proceed with a prospective clinical study. Secondly, the biological functions of the CpG sites in the model remain to be fully understood. Additionally, in the IV & M^−^ (*N* = 6) and IV & M^+^ (*N* = 10) subgroups, Kaplan–Meier curve analyses did not reveal any significant differences for PFI, DSS, and OS, likely due to the small sample size and advanced clinical stage of these patients, which limits definitive conclusions. Further external validation with larger datasets is required to confirm the model’s real-world applicability. Translating the 14-CpG model into clinical practice also presents challenges, particularly regarding cost. DNA methylation profiling may be hindered by its expense, especially in resource-limited environments, and while becoming more accessible, it may still be less feasible compared to traditional biomarkers such as hormone receptor and HER2 testing. Furthermore, the model needs validation across diverse patient populations, including different racial, ethnic, and geographical backgrounds, to ensure its generalizability and robustness. Finally, while the 14-CpG signature shows comparable or even superior predictive accuracy to established tools like Oncotype DX in certain subgroups, Oncotype DX is already widely integrated into clinical guidelines for assessing recurrence risk and guiding chemotherapy decisions. As such, the 14-CpG model requires further prospective validation and broader clinical integration before it can be directly compared with Oncotype DX in routine practice.

## Conclusion

In conclusion, our study has successfully developed and validated a novel 14-CpG-related DNA methylation signature that serves as a robust prognostic tool for BC. By using PFI as a clinical outcome, we provided a more sensitive measure for BC progression. This model identifies patient subgroups that could benefit from adjuvant chemotherapy, aiding personalized treatment decisions. Additionally, pathway analysis suggests potential targeted therapies for high-risk patients.

## Data Availability

Publicly available datasets were analyzed in this study. Data were obtained from TCGA and GEO datasets and are available at https://cancergenome.nih.gov/ (TCGA repository) and https://www.ncbi.nlm.nih.gov/geo/ with accession numbers: GSE22249, GSE66695, GSE22249, GSE66695, GPL8490, GPL13534.

## Footnotes

^1^ https://www.cancerrxgene.org

## Glossary

AJCC: American Joint Committee on Cancer
AUC: Area under the curve
BC: Breast cancer
CI: Confidence intervals
DGEs: Differential gene expressions
DOC: Distance on curve
DSS: Disease specific survival
ER: Estrogen receptors
GDSC: Genomics of Drug Sensitivity in Cancer
GDC: The Genomic Data Commons
GEO: The Gene Expression Omnibus
HER2: Human epidermal growth factor receptor 2
HR: Hazard ratio
KM: Kaplan–Meier
PFI: Progression free interval
PR: Progesterone receptors
OS: Overall survival
ROC: Receiver operating characteristic
TNBC: Triple-negative breast cancer
TCGA: The Cancer Genome Atlas
TNBC: Triple-negative breast cancer
TNM: T, tumor size, N, lymph node status, M, metastasis status
